# Child self-care autonomy in health (scale for parents): development, internal structure, and sex/age correlates

**DOI:** 10.3389/fpsyg.2023.1243400

**Published:** 2023-08-24

**Authors:** Oxana Mikhaylova, Anastasia Bochkor, Polina Osipova, Denis Popov, Maria Chepeleva, Evgenia Rybakova

**Affiliations:** ^1^Center for Contemporary Childhood Research, HSE University, Moscow, Russia; ^2^Department for Social Institutions Analysis, HSE University, Moscow, Russia; ^3^Laboratory for Psychology of Social Inequality, HSE University, Moscow, Russia; ^4^Centre for Institutional Research, HSE University, Moscow, Russia; ^5^Laboratory for Sports Studies, HSE University, Moscow, Russia; ^6^Centre for Student Academic Development, HSE University, Moscow, Russia; ^7^Department for Social Insitutions Analysis, HSE University, Moscow, Russia; ^8^Center for Sociocultural Research at HSE University, Moscow, Russia

**Keywords:** self-care autonomy in health, autonomy, autonomy development, parent–child relationships, children, scaling

## Abstract

Autonomy in self-care practices in the health sphere is a critical characteristic for the survival of humans throughout the life span. Notably, however, the current literature lacks psychometrically sound instruments that measure this phenomenon among children without diagnosed chronic health conditions. The purpose of the present exploratory study was to develop, test, and provide data regarding the reliability and validity of the Child Self-Care Autonomy in Health (CSAH) scale. The piloted version of the CSAH yielded an 11-item instrument designed to reflect the parent’s perspective in measuring the extent of autonomy in self-care actions related to health for a child, whether diagnosed with a chronic illness or not. Data were collected through an online survey of a non-random sample of Russian-speaking parents currently residing in Russia (*N* = 349). The analysis focused on scale structure via principal component analysis and age/sex associations. The proposed CSAH may be of interest to social workers, health professionals, and parents seeking to ascertain schoolers’ autonomy in self-care practices in the health sphere and support building a stronger self-care mindset.

## 1. Introduction

The current psychological literature typically associates increasing maturity with the development of individual responsibility for self-functioning in various life spheres, including health (Bröder et al., 2020; Radez et al., 2021; Magnusson et al., 2022; Renwick et al., 2022). For the purposes of this discussion, self-care in the health sphere refers to a complex of practices intended to maintain, monitor, and manage personal health conditions (Riegel et al., 2012).

The measurement of self-care behaviors in the health sphere is vital for children in all health conditions; for example, they offer the possibility of forming normative expectations based on sex and age since such measurements indicate the general well-being of the child population at the country level (Abbas et al., 2022). Furthermore, sex-and age-based differences in self-care practices may uncover the existence of age-and gender-related stereotypes inherent to the investigated culture. For instance, in collectivist cultures (which tend to emphasize collective over personal values), research has revealed that male adults experience more difficulty in adhering to healthy nutrition without spousal and whole-family support compared to their female counterparts because the former are not usually involved in cooking and meal serving; men also tend to assume that women should assist them in self-care and express the idea that women should support their husbands in achieving a healthy lifestyle (Lukman et al., 2020; Osokpo and Riegel, 2021).

In addition, data acquired in a specific country concerning health-related self-care practices among children without chronic conditions may be employed to target interventions more precisely (Dawson et al., 2022; Hopkins and Narasimhan, 2022). Screening for self-care behaviors and evaluating those who engage in minimal as well as excessive self-care at a certain age and for a certain sex can highlight individuals in need of a novel self-care intervention within a particular country, district, or school. Lastly, on the individual level, attentive parents desiring insight into their child’s health-focused behavior may benefit from a scale measuring self-care behaviors prior to consultation with a doctor in the form of a test that is relatively short and easy to use (Yerkes et al., 2021; Novoa et al., 2022; Pace et al., 2022).

Although the current literature offers many questionnaires assessing individual levels of self-care qualities among children with chronic conditions, no comparable instruments have been made available for the members of this population without acute or chronic conditions (Biagioli et al., 2022). The goal of this paper is to present a scale aimed at measuring self-care behaviors in the health sphere for individuals under age 18, regardless of the existence of diagnosed chronic illnesses^1^. An additional aim was to confirm whether this scale was age-and sex-specific.

The Adolescent Self-Care Autonomy in Health (CSAH) scale in the questionnaire this paper presents is intended for parents and was developed in the Russian context in 2022–2023 (the development period spanned Summer–Autumn 2022; data harvesting and piloting occurred in Spring 2023). Thus, future studies might explore adopting the scale for use in other countries. The current scale was initially applied in a qualitative pilot study that included interviews with 10 parents representing seven cities, while in the study’s later quantitative part, 349 parents participated. In Russia, almost 30–50% of people (the number differs from one data source to another) characterize their health as good or very good [Rostovskaya et al., 2021; Russian Public Opinion Research Center (WCIOM, 2021)]. Although no marked differences in health-focused self-care practices have been identified between sexes, women have sometimes demonstrated healthier lifestyles than men while simultaneously evaluating their health in worse terms than their male counterparts (Zhuravleva and Lakomova, 2019; Marconcin et al., 2021; Popova et al., 2021; Rostovskaya et al., 2021; WCIOM, 2021). Notably, these sex-related differences in health-based self-care were measured only in adults, and related data concerning Russian children is lacking. Research has also uncovered an interconnection between age and subjective health evaluation; specifically, increasing age has been correlated with a worsening self-evaluation of health (Popova et al., 2021; Rostovskaya et al., 2021; WCIOM, 2021). In terms of individuals under age 18, comparative studies have demonstrated that male and female Russian children were likely to report unhealthy lifestyles; furthermore, Russia scored the worst among other nations (Ruslyakova, 2015; Zhuravleva and Lakomova, 2019; Marconcin et al., 2021). As children mature, they consult their parents less when health-related situations arise and increasingly place their trust in doctors, peers, and media sources as they start to take on individual actions that affect health (Ruslyakova, 2015). In general, we hypothesized that our study might discover an interrelation between age and the level of autonomy in health-related self-care. We also anticipated that female children would be more attentive to their health than males.

## 2. Study self-care framework

Multiple terms have been employed to name the practices individuals use to influence their own health. Among the most popular have been self-care, self-organization, self-regulation, self-control, self-management, self-harm, coping, self-neglect, biohacking, and self-optimization (see Table 1 for various sources of these terms). These concepts can be roughly organized into groups based on an individual’s health status (having a chronic or acute health condition as opposed to no medically diagnosed or self-diagnosed illness), intentionality (intentional/unintentional), or the anticipated result of health influence (maintenance, improvement, deterioration; see Table 1 for examples). Furthermore, even within the mentioned terminology, conceptual clarity has not yet been established. For example, C. Godfrey et al. (2011) found 139 synonyms of the word “self-care” in scientific, practitioner-oriented, and lay speech. Taking dictionaries into consideration yields many additional closely related concepts (Matarese et al., 2018).

**Table 1:** Examples of conceptual varieties used to name personal health-influencing practices.

Concept	Individual health status	Intentionality	Anticipated result
Self-organization (Monsivais, 2005; Eppel-Meichlinger et al., 2022)	Chronic illness	Unintentional	Maintenance and improvement
Self-management (Audulv et al., 2016)	Chronic or acute	Intentional	Improvement
Self-care (Riegel et al., 2012)	Chronic or acute	Intentional	Maintenance and improvement
Self-optimization (Nehring and Röcke, 2023)	No condition	Intentional	Maintenance, improvement, and deterioration
Self-regulation (Hagger, 2010)	Chronic, acute, no condition	Intentional	Maintenance, improvement, and deterioration
Self-control (Gillebaart, 2018)	Chronic, acute, no condition	Intentional	Maintenance and improvement
Biohacking (Gangadharbatla, 2020)	No condition	Intentional	Improvement
Coping (Lazarus and Folkman, 1987)	Chronic, acute, no condition	Unintentional and intentional	Improvement
Self-harm (Tofthagen and Fagerstrøm, 2010)	Chronic, acute, no condition	Intentional	Maintenance and deterioration
Self-neglect (Lauder, 2001)	Chronic or acute	Unintentional and intentional	Deterioration

In this study, our interest lay in analyzing the practices that children employ in various health states when using them intentionally and with the aim to sustain but not necessarily improve or worsen health. Accordingly, we decided to apply the concept of “self-care” as initially suggested by Riegel et al. (2012). Thus, the term is defined as a complex of practices that are intended to maintain, monitor, and manage personal health conditions (Riegel et al., 2012; Biagioli et al., 2022). Self-care maintenance encompasses practices that help a person sustain emotional and physical stability, comprising (а) medical adherence, (b) treatment, (c) nutrition, (d) lifestyle, (e) prevention, and (f) familiarity with health-care services. Self-care monitoring is the process of observing signs and symptoms in terms of (a) clinical parameters and (b) physical manifestations. Lastly, self-care management means the response to signs and symptoms when they occur (consulting some sort of resource, human or otherwise).

In contrast to those who coined the original self-care concept, we extend the notion to include children who have no chronic or acute health conditions to fill the current gap concerning availability of scales capable of analyzing health-care practices performed by all individuals under age 18, including those without acute or chronic health issues (Biagioli et al., 2022). Our choice of Riegel and colleagues’ operational definition was motivated by the high level of psychometric parameters of the scales invented based on their definition (Biagioli et al., 2022).

Previous scales that scholars have applied to investigate health self-care practices in children have been aimed at children together with their close adults, such as parents (Patton et al., 2003; Modi et al., 2010; Giardini et al., 2011; Nazareth et al., 2018). However, in the case of the current investigation, the lack of existing empirical evidence made it problematic to state a specific age at which such a scale could be suggested to a minor without the need for a parent’s input.

In addition, it was difficult to estimate the possibility of observing sex-related differences in light of the lack of consensus on this aspect in the literature. Specifically, some researchers have noted differences in the intensity of self-care practices in the absence of external support or monitoring, as well as in the range of practices employed between sexes; contrariwise, other studies have not uncovered any significant relationship between sex and self-care (Dean, 1989; Masoumi and Shahhosseini, 2019; Stapley et al., 2020). The findings that demonstrate relationships between sex and self-care intensity are diverse (Masoumi and Shahhosseini, 2019). Some reports have asserted that women employ fewer self-care practices (Mansyur et al., 2015; Whipple et al., 2022), while contrasting studies have maintained that female participants adhered more closely to medical recommendations and performed better self-care than their male counterparts (Levin and Idler, 1983; Avedzi et al., 2018; Auttama et al., 2021). Concerning various self-caring behaviors, some researchers found that female participants adhered better than male participants in the area of nutrition, while the opposite results emerged in the case of physical exercise; however, evidence remains unclear regarding other behaviors (Mansyur et al., 2015; Avedzi et al., 2018; Auttama et al., 2021; Mohammadzadeh et al., 2021; Osokpo and Riegel, 2021; Whipple et al., 2022). Furthermore, the vast majority of previous studies were performed on specific populations with chronic conditions or included participants who were older than 20; therefore, the generalizability of these studies’ results to children is questionable. Regarding the association between age and self-care, it could be hypothesized that with increasing age, minors will act increasingly more autonomously from adults in self-care as their level of independence rises (Lee et al., 2021).

## 3. Methods

### 3.1. Scale development procedure

**Figure 1:**
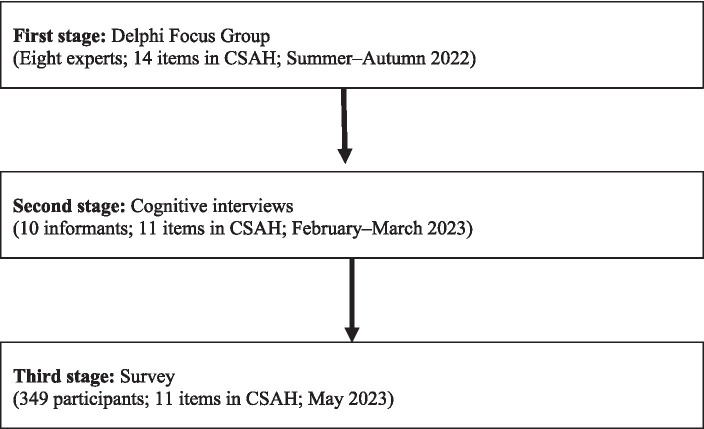
CSAH development process.

The questionnaire was developed in three stages (see Figure 1 for a summary of the process). The study was approved by HSE University ethical committee.

#### 3.1.1. Stage 1: Delphi focus group

In the first stage, we used the Delphi focus group method via online documents (Nasa et al., 2021) and offline research team meetings to collect information from eight child development scientists specializing in human developmental stages in Russia (referred to in the manuscript as “experts”). Specifically, the collected information targeted common self-care actions performed by children in Russia (Summer–Autumn 2022). From the information collected, we created a list of potential self-care practices that ultimately formed the study questionnaire for parents. The latter included 14 statements, along with a suggested ordinal 5-point scale to evaluate the degree of autonomy on the part of the minor in performing certain self-care actions as observed by the parent answering the questions. The points on the scale were as follows: “only with adult,” “if adult will explain the plan of action and will be nearby,” “if adult reminds and helps the child should the child request aid,” “adult is not involved but could help should the child request aid,” “child does everything on their own without reminders or help,” and “not applicable.” We chose to use a text-based rather than numeric scale to improve the validity of measurement; specifically, we wanted to ensure that parents put the same sense behind the actions in the questionnaire as we (the researchers) did.

#### 3.1.2. Stage 2: Cognitive interviews

The second part of the development process involved a two-stage piloting study that featured 10 cognitive interviews (Wolcott and Lobczowski, 2021) with parents (referred to as “informants” in this discussion) during February–March 2023. Participants were recruited using a purposive sampling method. Four of the researchers reached out to their personal networks to invite parents to take part in our research; in other words, we applied the snowball sampling method with four kernels, referring to the four researchers who recruited the current study’s informants. Our goal was to reach maximal variation in terms of the childs’ sex and grade. The resulting study sample included seven mothers and three fathers residing in seven Russian cities. Their offspring ranged in age from 8 to 18 (see Table 2). After the parents had submitted their responses to the questionnaires, four interviewers (three female and one male) aged under 22 years conducted face-to-face, semi-structured interviews in person and online. The entire research team created an interview guide organized into two major blocks (see Appendix). The first block was designed to clarify why participants chose a particular answer to the various questions, while the second block was dedicated to the analysis of the parent’s capacity to answer each particular question for their offspring. After the Stage 1 piloting, which involved three parents, four statements from the initial scale were excluded, and the measurement scale was corrected. As a result of observations made during the second stage (in which seven additional parents participated), no statements were excluded, though corrections were made, ultimately yielding 11 statements. The final version of the questionnaire is available in the Appendix.

**Table 2:** Cognitive interview participants.

Participant	Family role	Parent age	Offspring age	Grade	Offspring sex	Place of residence
1	Father	41	12	5	Male	Nizhny Novgorod
2	Mother	41	12	5	Male	Nizhny Novgorod
3	Father	46	11	4	Male	Kirov
4	Mother	35	8	2	Female	Tver
5	Mother	37	10	4	Female	Kirov
6	Father	38	11	5	Female	Moscow
7	Mother	45	14	8	Female	Nalchik
8	Mother	46	18	11	Female	Samara
9	Mother	46	14	7	Male	Moscow
10	Mother	52	17	11	Male	Yuzhno-Sakhalinsk

#### 3.1.3. Stage 3: Survey

In the third development stage, following the cognitive interview process conducted in May 2023, we distributed the questionnaire online via the social networks of the researchers. Specifically, we asked Russian-speaking parents currently living in Russia with one or more children attending school in Russia to participate in the survey. Respondents received no remuneration for participating.

The survey comprised two parts: (a) questions regarding the respondents’ sociodemographic characteristics (11 items, including parental age and sex, offspring age and sex, grade, place of residence, school type, and whether a child had chronic health conditions, such as asthma) and (b) the CSAH (11 statements). The full version is provided in the Appendix.

## 4. Results

The sample constituted 349 parents of Russian students (*M_parental age_* = 40, *SD _parental age_* = 5.74, min *_parental age_* = 21, max *_parental age_* = 66). Most of the respondents (94.3%) were mothers; fathers made up the remaining 5.7% of the sample. In terms of residence, 34% lived in megalopolises, while 30.9% lived in cities with a population ranging from 100,000 to 1 million citizens; 16.6% lived in cities with a population larger than 1 million citizens, 10% were living in villages, and 7.7% resided in cities with a population of less than 100,000 citizens or in urban settlements. The average age of schoolers whose parents took part in the study was 11.3 years, *SD* = 3.1, min = 6, max = 18. Slightly less than half of the childs (45.3%) were male, and slightly more than half (54.7%) were female. The largest portion (46.6%) studied in primary school (Grades 1–4); slightly fewer (36.4%) were in middle school (Grades 5–8), and the smallest percentage (17%) attended high school (Grades 9–11). A small number (15%) of the subjects had chronic health conditions, while 82.6% did not; however, 2.4% of the parents chose not to disclose such information about their offspring. Our self-care scale demonstrated a good level of reliability (*M* = 30.12, *SD* = 11.9, min = 8, max = 55, Cronbach’s alpha = 0.9). We calculated the mean individual scores and corresponding descriptive statistics by summing the responses ranging from 1 to 5 for each of the items. Additionally, the child could get 0 points for the item if the parent chose “inapplicable”; therefore, hypothetically, the total scores could have ranged from 0 to 55.

Two tasks were established for data analysis, as follows.

- Task 1 entailed analyzing the structure of the CSAH. As the questions for the self-care scale were formulated on the basis of a self-care conceptualization consisting of maintaining, monitoring, and managing personal health conditions, we anticipated that a three-component structure would emerge (H1). The small sample and exploratory design led us to use exploratory factor analysis to discern the structure of the questionnaire and put aside confirmatory factor analysis.
- Task 2 targeted analyzing the CSAH’s association with the respondents’ and their offspring’s demographics; additionally, we scrutinized scale distributions across age groups (primary, middle, and high school). Our second hypothesis posited that we would discover age (H2a) and sex (H2b) differences in scale distributions (our reasoning is described in the Introductory section). Statistical instruments included Spearman’s correlation, analysis of variance (ANOVA), and unpaired *t* tests, depending on the variable type.

All computations were made in the Statistical Package for the Social Sciences (SPSS). The following Results section is organized according to the corresponding task sections.

### 4.1. Analysis of questionnaire inner structure (principal component analysis)

**Figure 2:**
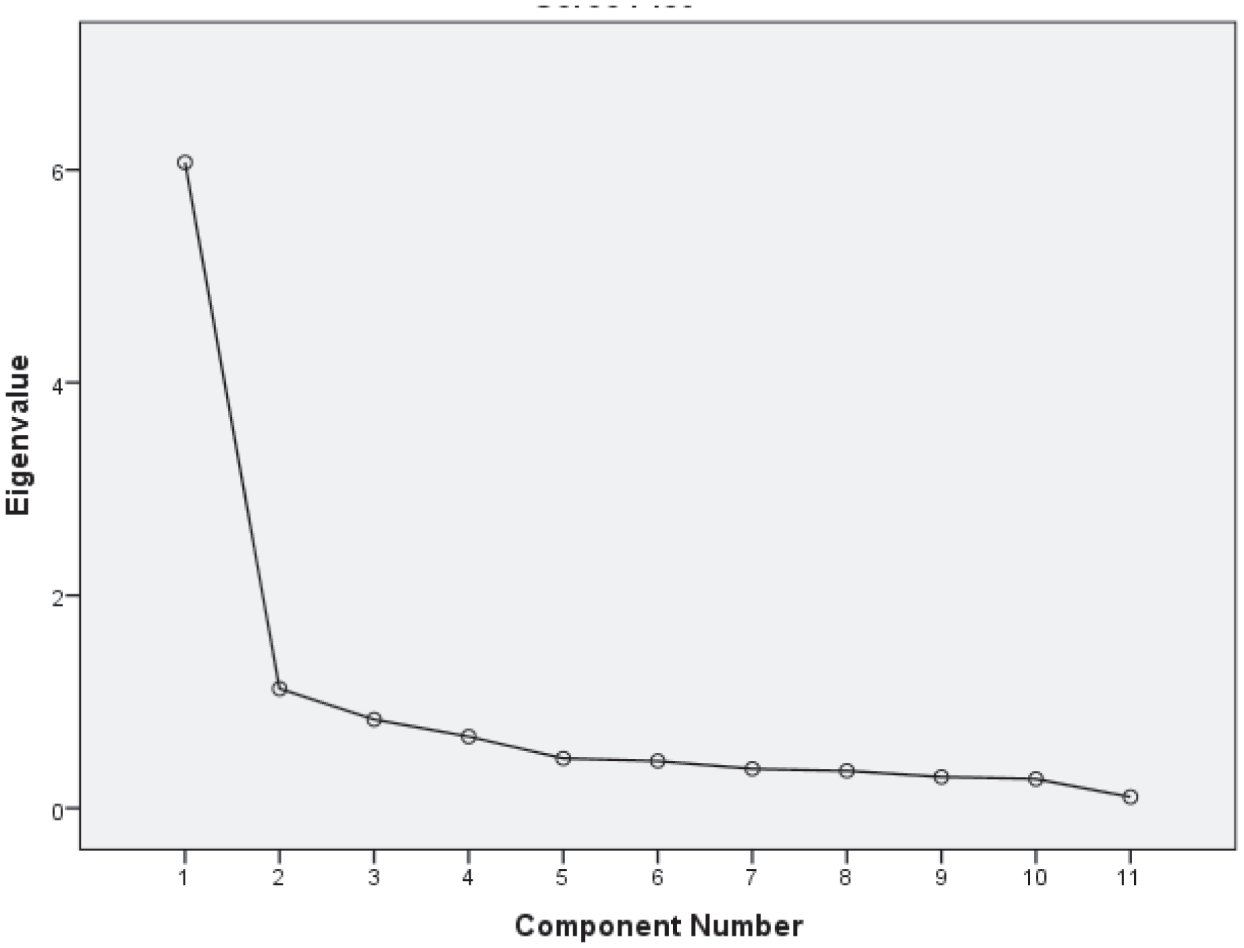
Scree plot for principal component analysis on CSAH.

The Kaiser-Meyer-Olkin measure of sampling adequacy (0.829) and Bartlett’s Test of Sphericity [*χ*^2^ (55) = 328,808, *p* < 0.00] demonstrated the possibility of performing exploratory factor analysis on the data (orthogonal rotation, “equamax”). Principal component analysis helped us identify two factors (Table 3), explaining 65% of the variance. Both factors had eigenvalues higher than 1 (Figure 2); therefore, we decided to accept this factorial decision.

**Table 3:** Factor loadings for CSAH.

Statements	Factor loadings	
	Component	
	Basic self-care	Advanced self-care
Соблюдает рекомендации врача в случае наличия хронических заболеваний или внезапного недомогания [Follows the doctor’s advice when dealing with chronic illnesses or a sudden malaise] (maintenance)	0.84	0.16
Идет к врачу в случае плохого физического самочувствия [Visits a doctor when experiencing deteriorating physical well-being] (management)	0.771	0.219
Устанавливает временные границы во время дня между отдыхом и учебой [Establishes specific time intervals during the day for both rest and study] (maintenance)	0.759	0.402
Измеряет температуру своего тела термометром в случае недомогания [Uses a thermometer to measure body temperature if feeling unwell] (management)	0.754	0.342
Лечит себя в случае легких недомоганий (порезы, заболело горло, болит голова, получил (−а) ожог) [Self-medicates for minor ailments, such as cuts, sore throat, headache, or burns] (management)	0.735	0.263
Одевается по погоде [Dresses appropriately considering the current weather conditions] (maintenance)	0.650	0.458
Умеет описывать свои симптомы в случае чувства недомогания [Possesses the ability to articulate symptoms when experiencing discomfort] (management)	0.606	0.323
Ежедневно отслеживает показатели своего здоровья, например, количество потребленных калорий [Regularly monitors health indicators daily, such as calorie intake] (monitoring)	0.262	0.837
Идет к психологу, в том числе школьному, в случае плохого психологического самочувствия [Seeks professional assistance from psychologists, including those available at school, when experiencing challenges to psychological well-being] (management)	0.194	0.811
Занимается физической активностью от 30 минут в день (физкультура, прогулки, спортивные секции) [Participates in physical activities for at least 30 min each day, which may include physical education, walking, or sports] (maintenance)	0.329	0.728
Питается сбалансировано и разнообразно (в рационе есть овощи, фрукты, крупы, мясо, рыба и т.д.) [Adopts a balanced, diverse diet that incorporates vegetables, fruits, grains, meat, fish, etc.] (maintenance)	0.371	0.665
Eigenvector values	6.07	1.12
Variance explained	55.182	10.200

Terms in parentheses represent the originally hypothesized (H1) components of self-care to which the statements should have belonged. The original statements from the scale in the Russian language are presented in square brackets.

The first factor comprised maintenance-and management-related actions connected with visiting a physician and adherence to medical recommendations, taking care if ill, being mindful of the weather conditions, and work–life balance. This factor explained 55% of scale variance. Based on the items included, we named the factor “Basic self-care,” referring to typical universal activities.

In contrast, the second factor included only monitoring, maintenance, and management actions that related to a healthy lifestyle (nutrition balancing, engaging in sports, self-tracking, and visiting a mental health specialist if needed) and explained 10% of scale variance. Based on the items included, we named the factor “Advanced self-care.”

### 4.2. Associations with age and sex

#### 4.2.1. Age

We applied several measures to examine the relationship between age and autonomy in self-care practices in the health sphere. First, we considered the child’s education level. Spearman’s rho coefficient between the level of education and CSAH was statistically significant (*p* < 0.001***, *n* = 295, *ρ* = 0.5). Since Russia features three levels of school education (primary, Grades 1–4; middle, 5–8; and high school, 9–11), we also used ANOVA (Table 4) to discover whether a child’s autonomy in self-care practices as reported by parents varied based on education level, revealing statistically significant differences. The level of effect *η*^2^ was moderate at >0.14 (Richardson, 2011). The level of autonomy was found to rise with the level of education.

**Table 4:** Means, standard deviations, and one-way analysis of variance in self-care autonomy in health sphere and level of education.

Measure	Primary		Middle		High		*F* (2)	*η*2
	*M*	*SD*	*M*	*SD*	*M*	*SD*		
Self-care autonomy in health	23.47	7.04	28.06	6.7	35.94	10.44	50.733***	0.26

***p < 0.001.

**Figure 3:**
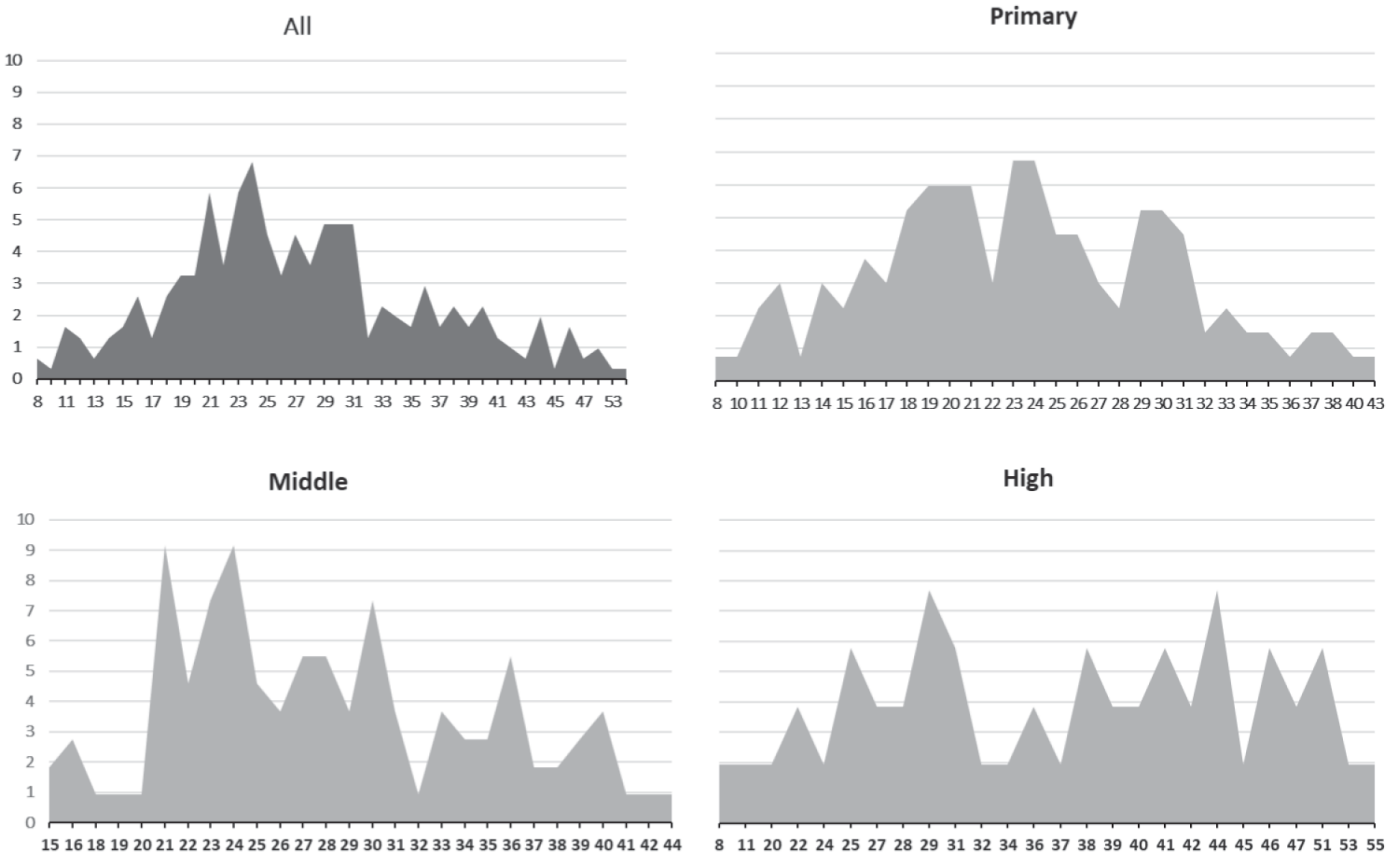
Percentage distributions for CSAH by educational level. *y*-axis = %. *x*-axis = points per CSAH in relation to education level.

To understand the parameters of self-care distributions within educational levels, we also calculated skewness and kurtosis (Table 5) and visualized percentages of points per scale (Figure 3). All distributions did not significantly differ from the normal distribution, as skewness and kurtosis did not surpass 2 (Jones, 1969). Furthermore, the measure of age employed the reported age of a child. Spearman’s rho correlation coefficient for child’s age and CSAH was statistically significant (*p* < 0.001***, *n* = 301, *ρ* = 0.51).

**Table 5:** Skewness, kurtosis, and standard errors divided according educational level.

Group	Skewness	*SE*	Kurtosis	*SE*
1–4	0.23	0.209	−0.31	0.41
5–8	0.30	0.231	−0.634	0.45
9–11	−0.446	0.330	−0.042	0.65
All	0.481	0.142	0.081	0.28

SE, standard error.

#### 4.2.2. Sex

The relationship between childrens’ sex and autonomy in self-care in the health sphere was analyzed using an unpaired *t* test. Female subjects were reported to have statistically significantly higher levels of autonomy in self-care compared to male counterparts (Table 6). However, the estimate of Cohen’s coefficient (*d* < 0.5) indicated only a small difference (Gignac and Szodorai, 2016).

**Table 6:** Means, standard deviations, and unpaired *t* test analysis of variance in self-care autonomy and sex.

Measure	Males		Females		*t* (298.948)	*p*	Cohen’s *d*
	*M*	*SD*	*M*	*SD*			
Self-care autonomy in health	25.74	8.95	28.96	8.74	−3.19	0.002	0.36

**Figure 4:**
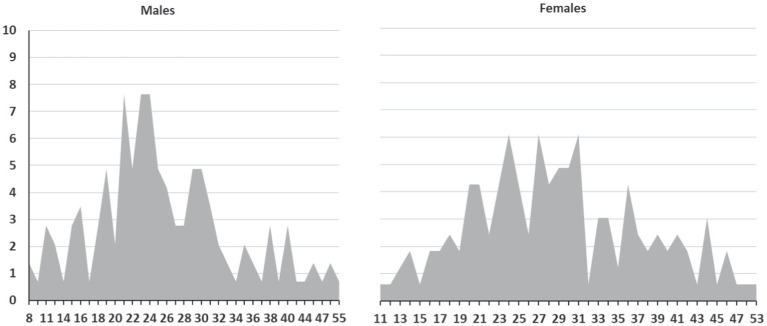
Percentage distributions for CSAH by sex. *y*-axis = %, *x*-axis = points per CSAH in relation to sex.

To understand the parameters of self-care distributions within sexes, we also calculated skewness and kurtosis (Table 7) and visualized percentages of points per scales (Figure 4). All distributions were not significantly different from the normal distribution, as skewness and kurtosis did not overrun 2.

**Table 7:** Skewness, kurtosis, and standard errors divided according to sex.

Sex	Skewness	*SE*	Kurtosis	*SE*
Male	0.71	0.20	0.76	0.4
Female	0.32	0.19	−0.40	0.37

SE, standard error.

## 5. Discussion

### 5.1. Components of self-care practices

Our findings contradict H1 (stating that the CSAH scale would reflect three components—management, monitoring, and maintenance; Riegel et al., 2012). Instead, the parents distinguished child autonomy in health self-care practices based on the complexity of the actions involved. Specifically, we discovered two factors behind self-care autonomy: “Basic self-care” and “Advanced self-care.”

Plausibly, our findings differ from the previous scales measuring self-care elaborated by scholars (Biagioli et al., 2022) primarily because our self-care scale was intended for application to children who were not diagnosed with chronic health conditions along with others who had such diagnoses. Supposedly, children who have not been diagnosed with chronic health conditions do not need to bother about health threats (whether isolated or multiple) every day, meaning that their life is not centered around health care as in the case of their peers with chronic health conditions. Therefore, it seems logical that their parents’ cognitive system includes a set of actions featuring both mandatory and beneficial but less urgent actions.

Also notable was that the parents’ responses distinguished between medical specialists dealing with physical health and mental health practitioners. Arguably, that distinction indicates that these parents considered mental health of secondary importance to physical health. This finding also corresponds with numerous polls conducted in Russia revealing the reluctance of most people to visit psychologists, especially individuals belonging to older generations who might have mental health issues and are not inclined to treat these issues as significant (WCIOM, 2022). Furthermore, our study could be interpreted as pointing to the possibility that parents and significant others convey this attitude toward mental health and mental health specialists in Russia to their children starting in childhood (Aguirre Velasco et al., 2020).

Furthermore, we find it necessary to emphasize that our findings reflect parental evaluations of self-care practices related to health. In this light, the division of practices between the two mentioned categories may not correspond to the reports regarding parents and their offspring in studies that have compared parents’ and childrens` views on home-based routines (Hou et al., 2019). These differences may arise from parental stereotypes, a lack of attention by parents to their offspring, and the adults’ desire to convey the best possible image of their children when participating in surveys.

Stereotypes may distort the measurement of child autonomy in self-care regarding health because parents may consider their offspring incompetent, reflecting ideas spread in the public sphere, especially in traditional media. For instance, major Russian television channels, especially federal ones, frequently portray children in Russia as very lazy and not capable of taking care of themselves. Vivid examples of reality shows demonstrating the non-capacity of children to be responsible for their bodies include *16 and Pregnant* (U-TV, 2023a), *Child from Hell* (U-TV, 2023b), and *Tomboys* (Friday, 2023). At the same time, parents who use social media may overestimate the capacity of their children for self-care because these media sources represent children as profound self-carers, making self-care an inescapable practice (Kaltefleiter and Alexander, 2019; Prasad and Mehendale, 2023).

Possibly, our findings might also have been affected by parental reflexivity and time spent with a child. For example, a parent who spends more time with their children might be more accurate in their estimates than one who spends less such time, especially if the former is attentive to their child’s self-care practices (Bylund et al., 2005). Furthermore, self-care, as it relates to body and intimacy, is a problematic sphere for a parent to monitor since being overly attentive to an child’s health status may be regarded as an invasion of privacy boundaries (Hernandez and Ebersole, 2022). In addition, parents’ ability to report certain health behaviors may vary. For instance, research indicates that risky health behaviors are typically underreported by parents (Bylund et al., 2005). Conversely, parental reports of sleep routines may be less distorted than those of their offspring (Mazza et al., 2020).

Another factor to consider is that answering survey questions may be a very sensitive practice for a parent. Thus, parents who worry about being stigmatized (i.e., considered “bad parents”) on the basis of their answers (Eaton et al., 2019) could provide elevated indications of their children’s self-care autonomy. Alternatively, they could consider such surveys as a measure of their children’s “goodness,” possibly motivating them to attempt to make the subject look better in the eyes of researchers by giving higher marks. Notably, in our surveys, more than half of the parents who provided answers to the questions were mothers. In this context, the literature reveals that mothers tend to be especially anxious about showing incompetence in parenting and being considered a bad parent, reflecting traditional gender role ideology that positions mothers as the primarily responsible parents for children’s well-being (Williamson et al., 2023). All of these factors might yield heightened scores on the questionnaire.

### 5.2. Age correlation with autonomy in self-care practices

Overall, we discovered that the older the subject was in terms of physical age and education level, the more the parents considered tis offspring as being autonomous in self-care actions in the health sphere. This finding is in tune with our assumption based on previous studies examining children with chronic health conditions (H2a), which similarly correlated increasing age with greater autonomy in self-care in relation to health (Biagioli et al., 2022).

Many factors may be involved in the explanation for such findings. For example, parents may be subject to age-related assumptions about their offspring, meaning an adult may believe that their child’s maturation should probably be accompanied by increasing autonomy in all actions, including those involving health (Miller, 2019). Parents may also cling to a sort of “real” order of development intimating that children become more responsible for their health with increasing age (Ruslyakova, 2015). In reality, the factors associated with increasingly responsible behavior remain complicated, including the roles played by nature, nurture, or both (Hargreaves et al., 2022). From an internal perspective, in the process of maturing, the body system develops and stabilizes, possibly making it easier for a child to pay attention to health with increasing maturity and understanding. From an external perspective, classes at school, media, parents, and significant others may teach students that practicing self-care is a vital skill that supports surviving and thriving. Conversely, cumulative effects of social influence and natural development may counter a child’s sound bodily knowledge and healthy development due to body image distortions, a low level of health literacy, bullying, and many other traumatic life events that commonly occur throughout childhood, adolescence, and young adulthood (Stapley et al., 2020; Lichner et al., 2021).

### 5.3. Sex association with autonomy in self-care practices

As we hypothesized in the context of Russia, we found a relationship between sex and autonomy in self-care practices (H2b) in that female subjects were arguably slightly more likely to engage in self-care than their male peers. These findings are in line with some papers reporting that women tended to exhibit healthier lifestyles than men, along with studies on self-care practices that analyzed sex differences (Levin and Idler, 1983; Avedzi et al., 2018; Auttama et al., 2021). From our viewpoint, the greater engagement in self-care reported in prior studies for female participants may reflect the more intense involvement of the girls’ mothers, whereas boys were found more likely to be controlled by their fathers (Poku et al., 2022). We also suppose that such a difference was found due to social assumptions about girls being more precise and obedient, along with expectations that girls and women are more concerned about beauty and health (Wood, 2023) than boys and men, for whom being too well-groomed could be considered unmasculine, especially in Russian culture (Gough and Novikova, 2020). These expectations may contribute to both a subject’s behavior and parental evaluations.

## 6. Strengths and practice, policy, and research implications

The primary strength of the CSAH is its brevity, which makes it easier to use in screening individuals, groups (grades), schools, country regions, and country-level comparisons. Screenings centered around the CSAH could also be combined with health literacy scales and classes presenting self-care activities that provide information about the self-care practices that are appropriate for children. Another valuable addition would entail training schoolers to perform these practices on their own since the lack of autonomy may arise from poorly preparing children to perform these activities independently and failing to provide adequate knowledge of the range of such activities.

The second strength of the CSAH is that it includes items analyzing the degree of self-care practices done by a child in terms of both mental and physical health, in contrast to previous scales, which tended to focus on either physical or mental health alone (Biagioli et al., 2022). Thus, the newly developed scale is more complete in terms of measuring self-care autonomy with regard to both the mental and physical spheres. Physical health and mental health are interconnected, making a combined assessment of autonomy in self-care practices in both spheres essential, as opposed to evaluating these aspects separately. For instance, changes in self-care autonomy in physical health, such as becoming more active in performing actions without external help, may also be accompanied by an increase in the individual’s expression of responsibility for mental health. Contrariwise, the extension of self-care autonomy in the area of physical health may introduce time constraints and other factors that impede an individual’s efforts to be more autonomous in mental health self-care.

The fact that the elaborated scale is sex-and age-specific makes it possible to use the CSAH in groups that are diverse in terms of sex and age ranges. For social policies, the findings of such screenings, including ours, may be used developing health-promoting programs. Future researchers who use the CSAH in screening may confirm our findings that female subjects and older children appear to be better in self-care autonomy in the health sphere according to their parents’ reports. In this case, steps may be taken to motivate boys to be more attentive to their health social campaigns, such as including more imaging of boys, along with guided discussions of the importance of self-care activities regardless of sex. Also, promoting autonomy and targeting health campaigns at students attending school, as well as their parents, may be vital, especially in the case of younger students. One aspect of such promotions should be to promote parents’ understanding that their children’s well-being depends on teaching minors to perform the necessary self-care activities on their own and resisting the temptation to be overly helpful.

## 7. Limitations and future recommendations

The present study has certain limitations that require consideration. Most importantly, we measured parental perceptions of child self-care behaviors related to health, raising the question of whether the children themselves might (or might not) comparably evaluate their self-care behavior. Future dyadic studies are needed to clarify this issue. Furthermore, our sample heavily favored mothers, thus obscuring whether the views we measured concerning child self-care would extend to fathers as well. Therefore, future research comparing different parents’ reports is vital. Additionally, since asking about more than two genders in studies conducted in Russia is not allowed, our analysis of the relationship between child sex and self-care measured this factor as a binary variable. However, an extension of the gender spectrum might have revealed divergent findings, such as the reports of studies in other countries demonstrating the likelihood that children with non-binary gender identities have faced certain stigmatizing social attitudes, potentially resulting in inadequate self-care and self-neglect (Martin-Storey and Baams, 2019). As Russia is not alone in forbidding the study of gender and multiple sexual identifies in children, comparable cases may be found in many Asian and African countries, such as Malaysia, Saudi Arabia, and Nigeria. Thus, delineating sex rather than only gender in regard to self-care activities may contribute valuable insight into the issue of juvenile health-related self-care because such measurements are more universally applicable across countries regardless of age (TGEU, 2022). Another limitation is that our study was conducted online, preventing parents who lacked access to the Internet from participating in the study and sharing their views. Accordingly, caution is needed when generalizing our results to parents with Internet access, and future studies should address parents who do not use the Internet in order to discern whether their evaluations of their children’s self-care practices might differ from those uncovered in our study. Also, as we have not tested convergent validity, the proposed scale requires further fine-tuning to make it applicable across counties and in demographically diverse samples. Finally, we must emphasize the necessity for further discussion of the division of self-care health behaviors in the context of the country where the study was conducted. Further research is needed, ideally applying mixed methods, to clarify whether our findings reflect only Russian culture and practice.

## 8. Conclusion

In this paper, we proposed and developed a scale to measure self-care autonomy in the health sphere for children who had no diagnosis related to chronic illnesses. Its quality was verified by a Delphi focus group, cognitive interviews, and an online survey. The final version of the questionnaire includes 11 items, has demonstrated good internal consistency, and features two factors (basic and advanced). In addition, the scale is correlated with age and sex. Specifically, older ages were associated with higher self-care autonomy in the health sphere, and children females were reported as having higher levels of self-care than their male counterparts. Additional research is needed that will examine larger samples to further analyze the proposed instrument’s psychometric traits, along with comparative inter-country studies that may aid uncovering the reasons behind sex-related differences.

## Data availability statement

The raw data supporting the conclusions of this article will be made available by the authors, without undue reservation.

## Ethics statement

The studies involving humans were approved by the HSE University (Higher School of Economics). The studies were conducted in accordance with the local legislation and institutional requirements. The participants provided their written informed consent to participate in this study.

## Author contributions

OM was responsible for elaborating the study design, writing the manuscript, conducting the literature review, and data analysis. AB took part in conducting cognitive interviews, recruiting survey respondents, and manuscript writing and managed the distribution of roles in the research team. PO, DP, and ER participated in conducting cognitive interviews, recruiting survey respondents, and writing the manuscript. MC recruited respondents for the survey and contributed to writing the manuscript. All authors contributed to the article and approved the submitted version.

## Funding

This research was supported by the Faculty of Social Sciences of HSE University (The program - supporting students research activities “Project Groups of Students” https://social.hse.ru/pg/).

## Conflict of interest

The authors declare that the research was conducted in the absence of any commercial or financial relationships that could be construed as a potential conflict of interest.

## Publisher’s note

All claims expressed in this article are solely those of the authors and do not necessarily represent those of their affiliated organizations, or those of the publisher, the editors and the reviewers. Any product that may be evaluated in this article, or claim that may be made by its manufacturer, is not guaranteed or endorsed by the publisher.

## Footnotes

^1^In this paper, this age category is referred to as “children.” We also use the term “under age 18” (accompanying designations such as “those,” “individuals,” or “people”) as a synonym to avoid repetition in phrasing.
