# Supplementary material for: Child self-care autonomy in health (scale for parents): development, internal structure, and sex/age correlates
Source: Front Psychol. 2023 Aug 24;14:1243400. doi: 10.3389/fpsyg.2023.1243400 (PMC10491485; doi:10.3389/fpsyg.2023.1243400)
Supplement: Supplementary file 1 [file Data_Sheet_1.docx]

Supplementary Material

Child Self-Care Autonomy in Health (Scale for Parents):

Development, Internal Structure, and Gender/Age Correlates

Oxana Mikhaylova, Anastasia Bochkor, Polina Osipova, Denis Popov, Maria Chepeleva, Rybakova Evgenia

*** Correspondence:**Oxana Mikhaylova
 oxanamikhailova@gmail.com

# PCA tables

Table 1. Descriptive statistics on items, N=349

| Statements | Mean | Std. Deviation |
| --- | --- | --- |
| 1. Uses a thermometer to measure body temperature if feeling unwell | 3,0943 | 1,54751 |
| 1. Possesses the ability to articulate symptoms when experiencing discomfort | 3,4906 | 1,47574 |
| 1. Visits a doctor when experiencing deteriorating physical well-being | 2,2642 | 1,53361 |
| 1. Self-medicates for minor ailments, such as cuts, sore throat, headache, or burns | 2,3019 | 1,40856 |
| 1. Participates in physical activities for at least 30 minutes each day, which may include physical education, walking, or sports | 3,1887 | 1,38754 |
| 1. Adopts a balanced, diverse diet that incorporates vegetables, fruits, grains, meat, fish, etc. | 2,4151 | 1,27753 |
| 1. Establishes specific time intervals during the day for both rest and study | 2,9623 | 1,58068 |
| 1. Dresses appropriately considering the current weather conditions | 3,2830 | 1,39197 |
| 1. Regularly monitors health indicators daily, such as calorie intake | 2,3396 | 1,50567 |
| 1. Follows the doctor’s advice when dealing with chronic illnesses or a sudden malaise | 2,6226 | 1,43079 |
| 1. Seeks professional assistance from psychologists, including those available at school, when experiencing challenges to psychological well-being | 2,3774 | 1,54704 |

Table 2. Correlations between statements, N=349.

| **Statement** | **1** | **2** | **3** | **4** | **5** | **6** | **7** | **8** | **9** | **10** | **11** |
| --- | --- | --- | --- | --- | --- | --- | --- | --- | --- | --- | --- |
| 1. Uses a thermometer to measure body temperature if feeling unwell | - | - | - | - | - | - | - | - | - | - | - |
| 1. Possesses the ability to articulate symptoms when experiencing discomfort | 0,569*** | - | - | - | - | - | - | - | - | - | - |
| 1. Visits a doctor when experiencing deteriorating physical well-being | 0,581*** | 0,468*** | - | - | - | - | - | - | - | - | - |
| 1. Self-medicates for minor ailments, such as cuts, sore throat, headache, or burns | 0,534*** | 0,344** | 0,577*** | - | - | - | - | - | - | - | - |
| 1. Participates in physical activities for at least 30 minutes each day, which may include physical education, walking, or sports | 0,583*** | 0,517*** | 0,283* | 0,393** | - | - | - | - | - | - | - |
| 1. Adopts a balanced, diverse diet that incorporates vegetables, fruits, grains, meat, fish, etc. | 0,427** | 0,359** | 0,394** | 0,517*** | 0,563*** | - | - | - | - | - | - |
| 1. Establishes specific time intervals during the day for both rest and study | 0,748*** | 0,593*** | 0,567*** | 0,627*** | 0,486*** | 0,494*** | - | - | - | - | - |
| 1. Dresses appropriately considering the current weather conditions | 0,630*** | 0,521*** | 0,505*** | 0,554*** | 0,519*** | 0,560*** | 0,643*** | - | - | - | - |
| 1. Regularly monitors health indicators daily, such as calorie intake | 0,423** | 0,348** | 0,518*** | 0,431** | 0,567*** | 0,575*** | 0,563*** | 0,513*** | - | - | - |
| 1. Follows the doctor’s advice when dealing with chronic illnesses or a sudden malaise | 0,572*** | 0,454*** | 0,686*** | 0,640*** | 0,434*** | 0,456** | 0,623*** | 0,557*** | 0,355*** | - | - |
| 1. Seeks professional assistance from psychologists, including those available at school, when experiencing challenges to psychological well-being | 0,411** | 0,355** | 0,419** | 0,370** | 0,513*** | 0,454*** | 0,470*** | 0,432** | 0,695*** | 0,344** | - |

* = p<.05, ** = p<.01, *** = p<.001

Table 3. Communalities, N=349.

| **Statements** | **Initial** | **Extraction** |
| --- | --- | --- |
| Uses a thermometer to measure body temperature if feeling unwell | 1,000 | 0,686 |
| Possesses the ability to articulate symptoms when experiencing discomfort | 1,000 | 0,472 |
| Visits a doctor when experiencing deteriorating physical well-being | 1,000 | 0,642 |
| Self-medicates for minor ailments, such as cuts, sore throat, headache, or burns | 1,000 | 0,610 |
| Participates in physical activities for at least 30 minutes each day, which may include physical education, walking, or sports | 1,000 | 0,638 |
| Adopts a balanced, diverse diet that incorporates vegetables, fruits, grains, meat, fish, etc. | 1,000 | 0,580 |
| Establishes specific time intervals during the day for both rest and study | 1,000 | 0,738 |
| Dresses appropriately considering the current weather conditions | 1,000 | 0,633 |
| Regularly monitors health indicators daily, such as calorie intake | 1,000 | 0,769 |
| Follows the doctor’s advice when dealing with chronic illnesses or a sudden malaise | 1,000 | 0,730 |
| Seeks professional assistance from psychologists, including those available at school, when experiencing challenges to psychological well-being | 1,000 | 0,695 |
| Extraction Method: Principal Component Analysis. | | |

Table 4. Variance explained, N=349.

| **Component** | **Initial Eigenvalues** | | | **Extraction Sums of Squared Loadings** | | | **Rotation Sums of Squared Loadings** | | |
| --- | --- | --- | --- | --- | --- | --- | --- | --- | --- |
|  | **Total** | **% of Variance** | **Cumulative %** | **Total** | **% of Variance** | **Cumulative %** | **Total** | **% of Variance** | **Cumulative %** |
| 1 | 6,070 | 55,182 | 55,182 | 6,070 | 55,182 | 55,182 | 4,126 | 37,508 | 37,508 |
| 2 | 1,122 | 10,200 | 65,382 | 1,122 | 10,200 | 65,382 | 3,066 | 27,874 | 65,382 |
| 3 | 0,833 | 7,574 | 72,956 |  |  |  |  |  |  |
| 4 | 0,673 | 6,115 | 79,071 |  |  |  |  |  |  |
| 5 | 0,468 | 4,253 | 83,323 |  |  |  |  |  |  |
| 6 | 0,443 | 4,028 | 87,351 |  |  |  |  |  |  |
| 7 | 0,369 | 3,354 | 90,705 |  |  |  |  |  |  |
| 8 | 0,350 | 3,181 | 93,886 |  |  |  |  |  |  |
| 9 | 0,293 | 2,667 | 96,553 |  |  |  |  |  |  |
| 10 | 0,274 | 2,493 | 99,046 |  |  |  |  |  |  |
| 11 | 0,105 | 0,954 | 100,000 |  |  |  |  |  |  |
| Extraction Method: Principal Component Analysis. | | | | | | | | | |

Table 5. Component Transformation Matrix N=349.

| **Component** | **1** | **2** |
| --- | --- | --- |
| **1** | 0,779 | 0,627 |
| **2** | -0,627 | 0,779 |
| Extraction Method: Principal Component Analysis.  Rotation Method: Equamax with Kaiser Normalization. | | |

# Questionnaire (English Version)

Good afternoon!

As psychologists and sociologists of the HSE Center for Contemporary Childhood Research, we are conducting a survey on how children exercise self-care autonomy in the area of health maintenance. After answering 11 general questions, you will be presented with two additional questionnaires that present a series of statements for you to consider. In the first questionnaire, which has 11 statements, we will ask you to indicate the degree to which your child performs the described actions independently. In the second questionnaire, which has 10 statements, we will ask you to evaluate how often you yourself perform the listed actions. The data you provide will be completely confidential and will be analyzed in a consolidated form. Please try to answer as honestly as possible. Thank you for your interest in our research!

Filling out the questionnaire will take about 10 minutes.

Please fill out the following brief information-gathering section about you and your child before completing the questionnaire. If you have several children attending school, fill in the information for the eldest child. Thanks!

***Part 1***

1. Your age (specify the number of full years)
2. Your gender

- Male
- Female

1. Age of your child (specify the number of full years)
2. Gender of your child

- Male
- Female

1. In what class does your child study (for example, 1b or 4b, etc.)?
2. The type of settlement where you currently live

- A village
- A city with a population of up to 100,000 citizens or an urban-type settlement
- A city with a population of 100,000 to 1 million citizens
- A city with a population of over 1 million citizens
- A megalopolis

1. Your child’s school is...

- Located in the same location where you live
- Located in another location in your region
- Located in another region

1. The type of settlement where your child’s school is located

- A village
- A city with a population of up to 100,000 citizens or an urban-type settlement
- A city with a population of 100,000 to 1 million citizens
- A city with a population of over 1 million citizens
- A megalopolis

1. The type of school your child attends

- Municipal school
- Private school
- My child is homeschooled.

1. The education format of the school your child goes to

- Elementary secondary school
- Primary secondary school
- Secondary school
- Gymnasium
- Lyceum
- Secondary school with in-depth study of individual subjects
- Progymnasium (primary school with a preschool department for older preschool children)
- Primary school with preschool groups (“primary school-kindergarten”)
- Educational complex
- Evening (shift) secondary school
- Boarding school

1. Does your child have any chronic diseases (e.g., bronchitis, asthma, diabetes mellitus)

- Yes
- No
- Not sure

***Part 2***

Please rate on the provided scale how independently your child performs these actions. Please focus your answers on the child whose information was provided on the first page.

1. Uses a thermometer to measure body temperature if feeling unwell

- Only with adult
- If adult will explain the plan of action and will be nearby
- If adult reminds and helps the child should the child request aid
- Adult is not involved but could help should the child request aid
- Child does everything on their own without reminders or help
- Not applicable

1. Participates in physical activities for at least 30 minutes each day, which may include physical education, walking, or sports

- Only with adult
- If adult will explain the plan of action and will be nearby
- If adult reminds and helps the child should the child request aid
- Adult is not involved but could help should the child request aid
- Child does everything on their own without reminders or help
- Not applicable

1. Dresses appropriately considering the current weather conditions

- Only with adult
- If adult will explain the plan of action and will be nearby
- If adult reminds and helps the child should the child request aid
- Adult is not involved but could help should the child request aid
- Child does everything on their own without reminders or help
- Not applicable

1. Self-medicates for minor ailments, such as cuts, sore throat, headache, or burns

- Only with adult
- If adult will explain the plan of action and will be nearby
- If adult reminds and helps the child should the child request aid
- Adult is not involved but could help should the child request aid
- Child does everything on their own without reminders or help
- Not applicable

1. Possesses the ability to articulate symptoms when experiencing discomfort

- Only with adult
- If adult will explain the plan of action and will be nearby
- If adult reminds and helps the child should the child request aid
- Adult is not involved but could help should the child request aid
- Child does everything on their own without reminders or help
- Not applicable

1. Follows the doctor’s advice when dealing with chronic illnesses or a sudden malaise

- Only with adult
- If adult will explain the plan of action and will be nearby
- If adult reminds and helps the child should the child request aid
- Adult is not involved but could help should the child request aid
- Child does everything on their own without reminders or help
- Not applicable

1. Seeks professional assistance from psychologists, including those available at school, when experiencing challenges to psychological well-being

- Only with adult
- If adult will explain the plan of action and will be nearby
- If adult reminds and helps the child should the child request aid
- Adult is not involved but could help should the child request aid
- Child does everything on their own without reminders or help
- Not applicable

1. Regularly monitors health indicators daily, such as calorie intake

- Only with adult
- If adult will explain the plan of action and will be nearby
- If adult reminds and helps the child should the child request aid
- Adult is not involved but could help should the child request aid
- Child does everything on their own without reminders or help
- Not applicable

1. Adopts a balanced and diverse diet that incorporates vegetables, fruits, grains, meat, fish, etc.

- Only with adult
- If adult will explain the plan of action and will be nearby
- If adult reminds and helps the child should the child request aid
- Adult is not involved but could help should the child request aid
- Child does everything on their own without reminders or help
- Not applicable

1. Establishes specific time intervals during the day for both rest and study

- Only with adult
- If adult will explain the plan of action and will be nearby
- If adult reminds and helps the child should the child request aid
- Adult is not involved but could help should the child request aid
- Child does everything on their own without reminders or help
- Not applicable

1. Visits a doctor when experiencing deteriorating physical well-being

- Only with adult
- If adult will explain the plan of action and will be nearby
- If adult reminds and helps the child should the child request aid
- Adult is not involved but could help should the child request aid
- Child does everything on their own without reminders or help
- Not applicable

***Part 3***

Please rate on the provided scale how often you perform the following actions.

1. I additionally help my child with those learning skills that are difficult to attain at school

- Almost never
- Rarely
- Sometimes
- Often
- Almost always
- It is difficult to answer

1. I assist my child with homework

- Almost never
- Rarely
- Sometimes
- Often
- Almost always
- It is difficult to answer

1. I tell my child how school helped me with my future life

- Almost never
- Rarely
- Sometimes
- Often
- Almost always
- It is difficult to answer

1. I discuss my child’s school life with close people

- Almost never
- Rarely
- Sometimes
- Often
- Almost always
- It is difficult to answer

1. I provide my child with opportunities to search for information about higher education (for example, regarding higher education institutions and career opportunities)

- Almost never
- Rarely
- Sometimes
- Often
- Almost always
- It is difficult to answer

1. I’m talking to my child about what their life will be like after graduation

- Almost never
- Rarely
- Sometimes
- Often
- Almost always
- It is difficult to answer

1. I encourage my child to invite his friends to our house

- Almost never
- Rarely
- Sometimes
- Often
- Almost always
- It is difficult to answer

1. I offer to help my child with homework

- Almost never
- Rarely
- Sometimes
- Often
- Almost always
- It is difficult to answer

1. I talk to my child about career or professional trajectories in which they may be interested

- Almost never
- Rarely
- Sometimes
- Often
- Almost always
- It is difficult to answer

1. I tell my child about my school experience

- Almost never
- Rarely
- Sometimes
- Often
- Almost always
- It is difficult to answer

***Part 4***

If you have comments on the questionnaire or specific questions, please leave them below.

# Questionnaire (Russian version)

Добрый день!

Мы, психологи и социологи Центра Исследований Современного Детства НИУ ВШЭ, проводим опрос, посвященный детской самостоятельности в области заботы о здоровье. После 11 общих вопросов вам будут представлены две анкеты: из 11 и 10 утверждений. В первой мы попросим вас указать степень, в которой ваш ребенок самостоятельно выполняет описанные действия. Во второй мы попросим вас оценить, насколько часто вы сами выполняете перечисленные действия. Предоставленные вами данные полностью конфиденциальны и будут анализироваться в обобщенном виде. Пожалуйста, постарайтесь отвечать максимально честно. Благодарим за интерес к нашему исследованию!

Заполнение анкеты займет примерно 10 минут.

Пожалуйста, перед прохождением опросника заполните краткую информацию о вас и вашем ребенке-школьнике. Если у вас несколько детей, посещающих школу, заполняйте информацию о старшем ребенке. Спасибо!

***Блок 1***

1. Ваш возраст (укажите число полных лет)
2. Ваш пол

- Мужской
- Женский

1. Возраст вашего ребенка (укажите число)
2. Пол вашего ребенка

- Мужской
- Женский

1. В каком классе учится ваш ребенок (например, 1б или 4в и т.д.)
2. Тип населенного пункта, где вы на данный момент проживаете

- Село
- Город с населением до 100 тыс. жителей или поселок городского типа
- Город с населением от 100 до 1 млн. жителей
- Город с населением свыше 1 млн. жителей
- Мегаполис

1. Школа вашего ребенка...

- Расположена в том же населенном пункте, в котором вы проживаете
- Расположена в другом населенном пункте вашего региона
- Расположена в другом регионе

1. Тип населенного пункта, в котором находится школа вашего ребенка

- Село
- Город с населением до 100 тыс. жителей или поселок городского типа
- Город с населением от 100 до 1 млн. жителей
- Город с населением свыше 1 млн. жителей
- Мегаполис

1. Тип школы, в которую ходит ваш ребенок

- Государственная (муниципальная)
- Частная
- Мой ребенок на домашнем, семейном обучении

1. Формат школы, в которую ходит ваш ребенок

- Начальная общеобразовательная школа
- Основная общеобразовательная школа
- Средняя общеобразовательная школа
- Гимназия
- Лицей
- Средняя общеобразовательная школа с углубленным изучением отдельных предметов
- Прогимназия (начальная школа с дошкольным отделением для детей старшего предшкольного возраста)
- Начальная школа с дошкольными группами («начальная школа-детский сад»)
- Образовательный комплекс
- Вечерняя (сменная) средняя общеобразовательная школа
- Школа-интернат

1. Скажите пожалуйста, имеются ли у вашего ребенка хронические заболевания (например, бронхит, астма, сахарный диабет)

- Да
- Нет
- Затрудняюсь ответить

***Блок 2***

Пожалуйста, оцените по шкале, насколько самостоятельно ваш ребенок выполняет данные действия. Пожалуйста, отвечайте о том ребенке, о котором указывали информацию на первой странице.

1. Измеряет температуру своего тела термометром в случае недомогания

- Только со взрослым
- Если взрослый сам опишет план действий и проследит за его выполнением
- Если взрослый напомнит и поможет по просьбе ребенка
- Взрослый НЕ включен в выполнение, но поможет по просьбе ребенка
- Ребенок делает сам без напоминаний и помощи
- Не возникало таких ситуаций

1. Занимается физической активностью от 30 минут в день (физкультура, прогулки, спортивные секции)

- Только со взрослым
- Если взрослый сам опишет план действий и проследит за его выполнением
- Если взрослый напомнит и поможет по просьбе ребенка
- Взрослый НЕ включен в выполнение, но поможет по просьбе ребенка
- Ребенок делает сам без напоминаний и помощи
- Не возникало таких ситуаций

1. Одевается по погоде

- Только со взрослым
- Если взрослый сам опишет план действий и проследит за его выполнением
- Если взрослый напомнит и поможет по просьбе ребенка
- Взрослый НЕ включен в выполнение, но поможет по просьбе ребенка
- Ребенок делает сам без напоминаний и помощи
- Не возникало таких ситуаций

1. Лечит себя в случае легких недомоганий (порезы, заболело горло, болит голова, получил(-а) ожог)

- Только со взрослым
- Если взрослый сам опишет план действий и проследит за его выполнением
- Если взрослый напомнит и поможет по просьбе ребенка
- Взрослый НЕ включен в выполнение, но поможет по просьбе ребенка
- Ребенок делает сам без напоминаний и помощи
- Не возникало таких ситуаций

1. Умеет описывать свои симптомы в случае чувства недомогания

- Только со взрослым
- Если взрослый сам опишет план действий и проследит за его выполнением
- Если взрослый напомнит и поможет по просьбе ребенка
- Взрослый НЕ включен в выполнение, но поможет по просьбе ребенка
- Ребенок делает сам без напоминаний и помощи
- Не возникало таких ситуаций

1. Соблюдает рекомендации врача в случае наличия хронических заболеваний или внезапного недомогания (диету/принимает лекарства/ полощет горло)

- Только со взрослым
- Если взрослый сам опишет план действий и проследит за его выполнением
- Если взрослый напомнит и поможет по просьбе ребенка
- Взрослый НЕ включен в выполнение, но поможет по просьбе ребенка
- Ребенок делает сам без напоминаний и помощи
- Не возникало таких ситуаций

1. Идет к психологу, в том числе школьному, в случае плохого психологического самочувствия

- Только со взрослым
- Если взрослый сам опишет план действий и проследит за его выполнением
- Если взрослый напомнит и поможет по просьбе ребенка
- Взрослый НЕ включен в выполнение, но поможет по просьбе ребенка
- Ребенок делает сам без напоминаний и помощи
- Не возникало таких ситуаций

1. Ежедневно отслеживает показатели своего здоровья, например, количество потребленных калорий, число сделанных шагов, объем выпитой воды, массу тела

- Только со взрослым
- Если взрослый сам опишет план действий и проследит за его выполнением
- Если взрослый напомнит и поможет по просьбе ребенка
- Взрослый НЕ включен в выполнение, но поможет по просьбе ребенка
- Ребенок делает сам без напоминаний и помощи
- Не возникало таких ситуаций

1. Питается сбалансировано и разнообразно (в рационе есть овощи, фрукты, крупы, мясо, рыба и т.д.)

- Только со взрослым
- Если взрослый сам опишет план действий и проследит за его выполнением
- Если взрослый напомнит и поможет по просьбе ребенка
- Взрослый НЕ включен в выполнение, но поможет по просьбе ребенка
- Ребенок делает сам без напоминаний и помощи
- Не возникало таких ситуаций

1. Устанавливает временные границы во время дня между отдыхом и учебой

- Только со взрослым
- Если взрослый сам опишет план действий и проследит за его выполнением
- Если взрослый напомнит и поможет по просьбе ребенка
- Взрослый НЕ включен в выполнение, но поможет по просьбе ребенка
- Ребенок делает сам без напоминаний и помощи
- Не возникало таких ситуаций

1. Идет к врачу в случае плохого физического самочувствия

- Только со взрослым
- Если взрослый сам опишет план действий и проследит за его выполнением
- Если взрослый напомнит и поможет по просьбе ребенка
- Взрослый НЕ включен в выполнение, но поможет по просьбе ребенка
- Ребенок делает сам без напоминаний и помощи
- Не возникало таких ситуаций

***Блок 3***

Оцените пожалуйста, как часто вы делаете следующие действия по следующей шкале.

1. Я дополнительно помогаю своему ребенку с теми учебными навыками, которые трудно даются в школе

- Почти никогда
- Редко
- Иногда
- Часто
- Почти всегда
- Затрудняюсь ответить

1. Я помогаю выполнять домашние задания своему ребенку

- Почти никогда
- Редко
- Иногда
- Часто
- Почти всегда
- Затрудняюсь ответить

1. Я рассказываю своему ребенку о том, как школа помогла мне в дальнейшей жизни

- Почти никогда
- Редко
- Иногда
- Часто
- Почти всегда
- Затрудняюсь ответить

1. Я обсуждаю школьную жизнь моего ребенка с близкими мне людьми

- Почти никогда
- Редко
- Иногда
- Часто
- Почти всегда
- Затрудняюсь ответить

1. Я обеспечиваю своему ребенку возможности по поиску информации о высшем образовании (например, о высших учебных заведениях, работе, на которую он может пойти)

- Почти никогда
- Редко
- Иногда
- Часто
- Почти всегда
- Затрудняюсь ответить

1. Я разговариваю со своим ребенком о том, какой будет его/ ее жизнь после окончания школы

- Почти никогда
- Редко
- Иногда
- Часто
- Почти всегда
- Затрудняюсь ответить

1. Я способствую тому, чтобы мой ребенок приглашал своих друзей к нам домой

- Почти никогда
- Редко
- Иногда
- Часто
- Почти всегда
- Затрудняюсь ответить

1. Я предлагаю помощь своему ребенку в выполнении домашних заданий

- Почти никогда
- Редко
- Иногда
- Часто
- Почти всегда
- Затрудняюсь ответить

1. Я разговариваю со своим ребенком о карьерах или профессиональных траекториях, в которых он может быть заинтересован

- Почти никогда
- Редко
- Иногда
- Часто
- Почти всегда
- Затрудняюсь ответить

1. Я рассказываю своему ребенку о том, как я учился/училась в школе

- Почти никогда
- Редко
- Иногда
- Часто
- Почти всегда
- Затрудняюсь ответить

***Блок 4***

Если у вас есть общие комментарии к анкете или отдельным вопросам, пожалуйста, оставьте их ниже.

# Guide (English Version)

*Questions that are asked for each question from the questionnaire:*

1. Please tell us why you chose this particular answer. How did you come to this answer—what were you thinking about/what did you remember?

*The following questions are asked if the respondent has not touched upon the topics in the narrative about how the person came to answer the question and in the example given for the situation.*

1. When was the last time there was a situation where ... *we repeat the context of the question and name the degree of independence of the child who was chosen*... Describe it. If you remember a more illustrative example, tell us about it, also.
2. Were all the words in the question clear to you? Did you have any difficulties reading the wording of the question? What was the question about?
3. Was some information confusing to you?
4. Did you doubt your answer? (If yes) If not this option, which one would you choose?

*Additional questions to some items in the questionnaire*

1. Measures body temperature with a thermometer in the case of malaise
2. Daily monitors health indicators, for example, the number of calories consumed, the number of steps taken, the amount of water drunk, body weight

*What is meant by health indicators? -> do the indicators suggested in the answer correspond to your understanding of what health indicators are?*

1. Follows the doctor’s recommendations in the case of chronic disease or a sudden malaise (dieting/taking medications/gargling)

*What is meant by “the doctor’s recommendations”?*

1. Dresses according to the weather
2. Goes to a psychologist in the case of challenges to psychological well-being
3. Eats in a balanced way (remains within the calorie norm for age and height; eats when feeling hungry; includes vegetables, fruits, cereals, meat, and fish in the diet)

*What does “balanced eating” mean to you?*

1. Heals themself in case of mild ailments (cuts, sore throat, headache, got a burn)

*What is meant by “a slight malaise”?*

1. Is able to describe symptoms when feeling unwell
2. Goes to the doctor when experiencing poor physical health

*What does “poor physical health” mean to you?*

1. Sets time limits during the day between rest and study

*How do you understand what it means to “set time limits”?*

1. Engages in physical activity for about 60 minutes a day

*What is “physical activity” in your understanding? When you answered the question, what kind of activity did you have in mind?*

**2 block of questions**

1. I discuss my child’s school life with family/friends.

*How exactly do you understand “school life”?*

1. I talk to my child about careers or professional trajectories in which they may be interested.

*How do you understand “career and professional trajectories”?*

1. I help or offer to help my child with homework.
2. I’m talking to my child about what their life will be like after graduation.
3. I tell my child about my studies at school.
4. I provide my child with opportunities to search for information about higher education (for example, regarding higher education institutions, a potential future job).

*What “search” situations come to your mind?*

1. I help my child with those learning skills that are difficult for them.

*How do you understand the phrase “learning skills”?*

1. I tell my child about how school helped me in later life.
2. I encourage my child to invite their friends to our home.

Final questions about the questionnaire as a whole:

1. How do you like the questionnaire—can you share your impressions? Was it difficult or easy to work with her?
2. The following questions are asked if the respondent has not touched upon them in the narrative about how he came to answer the question and in the task which intended to give the example of the situation.
3. What do you think about the answers that were offered to you? Was it convenient to answer them? Was there a feeling that none of the answers fit what you would have liked to choose?
4. Do you think your child would have answered these questions differently? Which ones, exactly? What areas of the child’s life do they cover?
5. When you answered the questionnaire questions, did you answer each question separately, or did you compare them with each other? Did your answers to some questions, in your opinion, affect the answers to other questions? Was there a feeling that there was a lack of structuring in the order of the questionnaire questions? (If yes) If we split the questions into thematic blocks, how exactly would it be more convenient for you?

The block “Communication of parents and children”:

1. How involved are you in the lives of your children? Can you assess how much you know about what is going on in their lives? What areas do you know more about? Which aspects are less familiar to you? How much do you know about your child’s daily routine?
2. How often do you spend time with your family?
3. How well do you think your children trust you?
4. Do you think parents in general can answer the questions discussed earlier for their children? Can their opinions differ from their children’s?

# Guide (Russian version)

*Вопросы, которые задаются к каждому вопросу из анкеты:*

Расскажите, пожалуйста, почему ты выбрал именно этот вариант ответа? Как ты пришел к такому ответу, о чем думал? / что вспоминал?

*Следующие вопросы задаются в том случае, если респондент не освятил их в нарративе о том, как он пришёл к ответу на вопрос и в примере ситуации.*

1. Когда в последний раз была ситуация, где … *повторяем контекст вопроса и называем степень самостоятельности ребенка, которая была выбрана..*. Опишите ее. Если помните более наглядный пример, расскажите о нем тоже.
2. Все ли слова в вопросе были тебе понятны? Были ли у тебя трудности с прочтением формулировки вопроса?  О чем был вопрос?
3. Может быть, какая-то информация показалась лишней/ какая-то информация вас сбивала?
4. Сомневался ли ты в своем ответе? (*Если да)* Если не этот вариант, то какой бы ты выбрал? Выбрал бы ты вариант “Затрудняюсь ответить”, если бы он был в анкете?

*Дополнительные вопросы к некоторым вариантам ответа:*

1. Ежедневно отслеживает показатели своего здоровья, например количество потребленных калорий, число сделанных шагов, объем выпитой воды, массу тела

- *Что понимается под показателями здоровья? -> предложенные в ответе показатели соответствуют вашему пониманию того, что такое показатели здоровья?*

1. Соблюдает рекомендации врача в случае наличия хронических заболеваний или внезапного недомогания  (диету/принимает лекарства/ полощет горло)

- *Что понимается под рекомендациями врача?*

1. Питается сбалансированно (находится в рамках нормы калорий для своего возраста и роста,  по чувству голода, в рационе есть овощи, фрукты, крупы, мясо рыба)

- *Что для вас значит "питается сбалансировано"?*

1. Лечит себя в случае легких недомоганий (порезы, заболело горло, болит голова, получил(-а) ожог)

- *Что понимается под легким недомоганием?*

1. Идет к врачу в случае плохого физического самочувствия

- Что вы понимаете под плохим физическим самочувствием?

1. Устанавливает временные границы во время дня между отдыхом и учебой

- Как вы понимаете, что означает “устанавливать временные границы”?

1. Занимается физической активностью около 60 минут в день

- Что такое “физическая активность” в вашем понимании? Когда вы отвечали на вопрос, какой вид активности вы имели в виду?

**2 блок вопросов**

1. Я обсуждаю школьную жизнь моего ребенка с семьей /друзьями

Что именно вы поняли под “Школьной жизнью”?

1. Я разговариваю со своим ребенком о карьерах или профессиональных траекториях, в которых он может быть заинтересован

- Как вы понимаете "карьерные и профессиональные траектории?"

1. Я обеспечиваю своему ребенку возможности по поиску информации о высшем образовании (например, о высших учебных заведениях, работе, на которую он может пойти)

- Какие ситуации "поиска" приходят вам на ум?

1. Я помогаю своему ребенку с теми учебными навыками, которые ему трудно даются

- Как вы понимаете словосочетание "учебные навыки?"

***Финальные вопросы об анкете в целом***

1. Как Вам анкета — ваши впечатления? Трудно или легко было с ней работать?

*Следующие вопросы задаются в том случае, если респондент не освятил их в нарративе о том, как он пришёл к ответу на вопрос и в примере ситуации.*

1. Что Вы думаете о вариантах ответов, которые Вам предлагались? Было ли удобно отвечать по ним? Было ли ощущение, что ни один из вариантов ответов не подходит под то, что Вы бы хотели выбрать?
2. Как вы считаете, ваш ребенок по-другому бы ответил на эти вопросы? На какие именно? Какие сферы жизнедеятельности ребенка они охватывают?
3. Когда Вы отвечали на вопросы анкеты, вы отвечали на каждый вопрос в отдельности или всё же сопоставляли их между собой? Повлияли ли Ваши ответы на одни вопросы, по Вашему мнению, на ответы на другие вопросы? Было ли ощущение, что не хватает структурированности в порядке расположения вопросов анкеты? *(Если да)* Если бы мы разбили вопросы по тематическим блокам, чем именно это было бы удобнее для вас?

***Блок «Связь родителей и детей»***:

1. Насколько Вы вовлечены в жизнь детей? Как Вы можете оценить, насколько Вы знаете о том, что происходит в их жизни? О каких сферах вы знаете лучше всего? О каких – хуже? Насколько вы знаете о повседневном режиме своего ребенка?
2. Как часто вы проводите время в кругу семьи?
3. Как Вы считаете, насколько дети Вам доверяют?
4. Считаете ли Вы, что родители могут отвечать на обсуждаемые ранее вопросы за своих детей? Их мнения могут расходиться?
